# A need to raise the bar — A systematic review of temporal trends in diagnostics for Japanese encephalitis virus infection, and perspectives for future research

**DOI:** 10.1016/j.ijid.2020.03.039

**Published:** 2020-06

**Authors:** Tehmina Bharucha, Freya M. Shearer, Manivanh Vongsouvath, Mayfong Mayxay, Xavier de Lamballerie, Paul N. Newton, Nicole Zitzmann, Ernest Gould, Audrey Dubot-Pérès

**Affiliations:** aDepartment of Biochemistry, University of Oxford, Oxford, UK; bLao-Oxford-Mahosot Hospital-Wellcome Trust-Research Unit, Mahosot Hospital, Vientiane, Laos; cModelling and Simulation Unit, Centre for Epidemiology and Biostatistics, Melbourne School of Population and Global Health, The University of Melbourne, Melbourne, Australia; dInstitute of Research and Education Development, University of Health Sciences, Vientiane, Laos; eCentre for Tropical Medicine & Global Health, Nuffield Department of Medicine, University of Oxford, Oxford, UK; fUnité des Virus Émergents (UVE: Aix-Marseille Univ-IRD 190-Inserm 1207-IHU Méditerranée Infection), Marseille, France

**Keywords:** Flaviviruses, Neurological infection, Diagnostics

## Abstract

•*Japanese encephalitis virus* (JEV) remains a leading cause of neurological infection in Asia.•A systematic review identified 20,212 published human cases of laboratory-confirmed JEV infections from 205 studies.•15,167 (75%) of cases were confirmed with the lowest confidence diagnostic test, i.e., level 3 or 4, or level 4.•Only 109 (53%) of the studies reported contemporaneous testing for dengue-specific antibodies.•A fundamental pre-requisite for the control of JE is lacking — that of a simple and specific diagnostic procedure that can be adapted for point-of-care tests and readily used throughout JE endemic regions of the world.

*Japanese encephalitis virus* (JEV) remains a leading cause of neurological infection in Asia.

A systematic review identified 20,212 published human cases of laboratory-confirmed JEV infections from 205 studies.

15,167 (75%) of cases were confirmed with the lowest confidence diagnostic test, i.e., level 3 or 4, or level 4.

Only 109 (53%) of the studies reported contemporaneous testing for dengue-specific antibodies.

A fundamental pre-requisite for the control of JE is lacking — that of a simple and specific diagnostic procedure that can be adapted for point-of-care tests and readily used throughout JE endemic regions of the world.

## Introduction

**Figure 1 fig0005:**
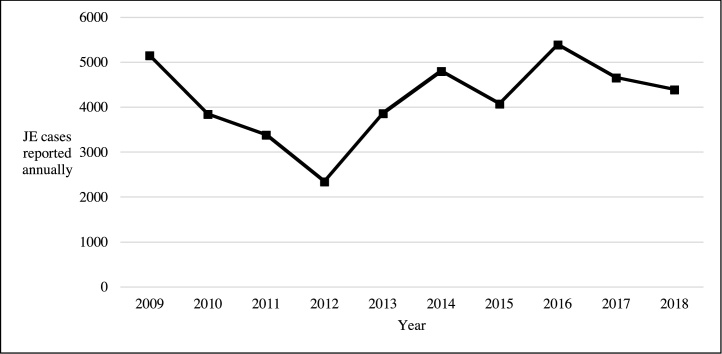
Number of JE cases reported annually over the last decade based on WHO/UNICEF surveillance (World Health Organization, 2019b). Data include probable* and laboratory-confirmed cases reported by JEV endemic countries. *WHO definition of a probable case (Solomon et al., 2008a) = A case that meets the clinical case definition for acute encephalitis syndrome (AES) that occurs in close geographical and temporal relationship to a laboratory-confirmed case of JE, in the context of an outbreak. Note that these data represent only reported cases, and are not considered to be an accurate representation of global JE incidence. The weaknesses of these data are discussed in the main text.

The mosquito-borne flavivirus Japanese encephalitis virus (JEV) accounts for an estimated 68,000 cases of Japanese encephalitis and 709,000 disability-adjusted life years annually (Fischer et al., 2008, Campbell et al., 2011). Japanese encephalitis virus (JEV) primarily affects children in rural areas when JEV-infected mosquitoes feed on humans rather than their primary amplifying hosts, pigs, or reservoir hosts, i.e., aquatic birds (Pearce et al., 2018). Sustained efforts from international agencies have supported the introduction of immunization programs into routine health control schedules in countries with endemic JEV transmission, Table 1 (Heffelfinger et al., 2017). The evidence suggests that vaccination has had an impact on JE incidence (Heffelfinger et al., 2017, Impoinvil et al., 2013, Yang et al., 2016, Ozawa et al., 2017, Upreti et al., 2017, Yu et al., 2018, Muniaraj and Rajamannar, 2019). However, JEV remains a leading cause of neurological infection in endemic countries, and the Joint World Health Organisation (WHO)/United Nations Children's Fund (UNICEF) surveillance data do not substantiate the improvements cited in the past ten years, with sustained numbers of reported patients over this period, see Figure 1.

**Table 1 tbl0005:** Country-specific data on the introduction of Japanese encephalitis virus vaccine in JEV endemic countries. Data adapted from the CDC report by Heffelfinger et al. 2017 and updated with WHO surveillance data (Heffelfinger et al., 2017, World Health Organization, 2017a, World Health Organization, 2019a).

Country	WHO region	Vaccine in schedule (2019)	JE immunization program	Year introduced subnationally	Year introduced nationally	Scheduled age (months) for vaccine	Vaccine used in immunisation program
Australia	WPRO	Yes	Risk areas: outer islands of Torres Straits	n/a	n/a	12	Live-recombinant
Bangladesh	SEARO	No	None	n/a	n/a	–	–
Bhutan	SEARO	No	None	n/a	n/a	–	–
Brunei Darussalam	WPRO	No	None	n/a	n/a	–	–
Cambodia	WPRO	Yes	National	2009	2015	9	Live-attenuated
People’s Republic of China	WPRO	Yes	National; excluding Qinghai, Tibet, Xinjiang, and Hong Kong which do not have endemic transmission	2003*	2008	8	Live-attenuated
DPR of Korea	SEARO	No	None; JEV vaccination campaign in 2016	n/a	n/a	–	–
India	SEARO	Yes	Subnational	2007	n/a	9–11	Live-attenuated
Indonesia	SEARO	Yes	Subnational: Bali	2018	n/a	–	–
Japan	WPRO	Yes	National	<2002	<2007	6	Inactivated vero cell derived
Lao PDR	WPRO	Yes	National	2013	2015	9–11	Live-attenuated
Malaysia	WPRO	Yes	Subnational: Sarawak and Sabah	2002	n/a	9	Live-recombinant
Myanmar	SEARO	Yes	National	n/a	2018	–	–
Nepal	SEARO	Yes	National	2007	2017	12	Live-attenuated
Pakistan	EMRO	No	None	n/a	n/a	–	–
Papua New Guinea	WPRO	No	None	n/a	n/a	–	–
Philippines	WPRO	Yes	Subnational: Regions I-III, and the Cordillera Administrative Region	2018	n/a	–	–
Republic of Korea	WPRO	Yes	National	n/a	<2002	12	Live-attenuated, Live-recombinant, Inactivated vero cell and mouse brain derived
Russian Federation	EURO	No	None	n/a	n/a	–	–
Singapore	WPRO	No	None	n/a	n/a	–	–
Sri Lanka	SEARO	Yes	National	2001	2011	12	Live-attenuated
Republic of China	WPRO	Yes	National	1963	1968	15	Inactivated mouse brain derived
Thailand	SEARO	Yes	National	n/a	<2002	12	Live-attenuated and Live-recombinant
Timor-Leste	SEARO	No	None	n/a	n/a	–	–
Vietnam	WPRO	Yes	National	<2002	2015	12	Inactivated mouse brain derived

* According to official WHO data, although it is acknowledged that the People’s Republic of China has performed widespread vaccination since 1971 (Gao et al., 2014). WPRO = Western Pacific Regional Office; SEARO = South-East Asia Regional Office.

JE cases reported to WHO/UNICEF have significant limitations. For example, increased awareness of the disease and access to laboratory capacity may contribute to increased case reporting. Conversely, surveillance data are likely to represent only a small proportion of patients (M’Ikanatha et al., 2013). This is particularly relevant for JE, occurring predominantly in rural areas lacking diagnostic capacity (Vaughn and Hoke, 1992). There are no rapid or point-of-care tests for JE in clinical use (Sengvilaipaseuth et al., 2017), and the WHO recommended standard diagnostic assay is an ELISA test that requires trained professionals, appropriate resources, and several hours for the results of the tests to be obtained (Western WHOROft, 2014). In a survey performed by WHO/UNICEF in 2017, 21 countries responded, of which 11 met the minimum surveillance standards (World Health Organization, 2018, World Health Organization, 2017b). Equally, there are problems of specificity of the most widely used diagnostic test, JE MAC-ELISA (Dubot-Peres et al., 2015). This is an increasing issue, with increasing endemicity of other flaviviruses and vaccination coverage.

The reasons for the persistence of JE as a public health problem are complex and multifactorial. A fundamental principle that must be kept in mind is that JE is a zoonotic infection; human immunization will never eradicate it in the natural environment and therefore sustained vaccination coverage is necessary. However, in countries that do have vaccination programs, they are not necessarily uniformly implemented nationwide, and in some areas, vaccine coverage is sub-optimal. While there are many reasons for inadequate coverage, this remains a neglected aspect of JE vaccination programs (Murhekar et al., 2017). Furthermore, immunization strategies are constrained by the absence of adequate diagnostic capacity to investigate the burden of disease, the impact of vaccination (Tandale et al., 2018), and the dynamic epidemiology of JE. For example, in common with other emerging arboviruses (Gould et al., 2017), JE has the propensity to emerge and become established in new geographical regions (Mackenzie et al., 2004). In recent years there have been increasingly frequent reports of cases in peri-urban and urban areas (Gould et al., 2017), as well as new regions such as Rajasthan, India.

Moreover, evidence for autochthonous transmission of JEV in Angola was recently reported (Simon-Loriere et al., 2017a). JEV RNA has also recently been detected in birds in Italy (Preziuso et al., 2018). This most likely represents the globally increased movement, via transportation, of animals and goods. Increased pig farming in urban areas of Asian countries also impacts on virus amplification. Finally, the live attenuated vaccine in widespread use is based on JEV genotype 3, even though in recent decades, there has been large-scale genotype displacement to genotype 1 (Wei et al., 2019), and evidence of detection of genotype 5 (Cao et al., 2016).

Accordingly, we performed a comprehensive review of the evolution of current diagnostic tests for JE. We tackled this by performing a systematic review of published laboratory-confirmed symptomatic cases of JE in humans, and extracted data on the laboratory procedures employed. We also appraised novel tests either under development or conceptually applicable for future diagnostic purposes. Data analysis informed our discussion on future perspectives for research.

## Methods

Searches were performed in the Web of Science and PubMed using the text word term 'Japanese encephalitis' up to 13th October 2019. The abstracts were reviewed, and a full text was obtained for those potentially containing information on human cases of JE in the English language. The full-text articles were then reviewed for those reporting symptomatic human cases of laboratory-confirmed JE. The search was limited to JE cases confirmed during the acute illness or hospitalization rather than seroprevalence, with geographic information at least to the country of onset of illness, and temporal information at least to the year of diagnosis. Data were extracted on details of the diagnostic confirmation of JE cases. A JE case was classified according to the confirmatory level (Fischer et al., 2008, Campbell et al., 2011, Pearce et al., 2018, Heffelfinger et al., 2017) developed from existing WHO and CDC criteria, where 1 provides the highest level of confidence based on the diagnostic test used, as illustrated in Table 2.

**Table 2 tbl0010:** Diagnostic criteria used to assess JE laboratory-confirmed patients.*

Level 1	JEV RNA detected in any specimen by RT-PCR.
	Virus isolation by inoculation of any specimen in cell culture or animal with characteristic cytopathic effect and confirmation by detection of JEV RNA or virus antigen.
	JEV virus antigen detected from brain tissue or CSF by immunofluorescence or immunohistochemistry
Level 2	Seroconversion or ≥ 4x rise in anti-JEV Ab by seroneutralization or detection of neutralizing antibody in CSF; Samples should be tested alongside other endemic flaviviruses (e.g., dengue viruses)
Level 3	Anti-JEV IgM detected in CSF; Samples should be tested alongside other endemic flaviviruses (e.g., dengue viruses)
	Seroconversion or 4x rise in anti-JEV Ab HI, CF, IFA, or seroconversion by ELISA; Samples should be tested alongside other endemic flaviviruses (e.g., dengue viruses).
Level 4	Anti-JEV IgM detected in serum in one sample (acute/convalescent), or seroneutralisation tested in one sample or single high titer HI/CF/IFA; Samples must be tested alongside other endemic flaviviruses (e.g., dengue viruses)

RT-PCR = reverse transcription-polymerase chain reaction; RNA = ribonucleic acid; CSF = cerebrospinal fluid; Ab = antibody; ELISA = enzyme-linked immunosorbent assay; HI = haemagglutination inhibition; CF = complement fixation test; IFA = indirect immunofluorescence assay.

* Confirmation of JE is categorized into levels 1-4 based on existing WHO and CDC criteria, such that level 1 provides the highest level of confidence.

## Results

**Figure 2 fig0010:**
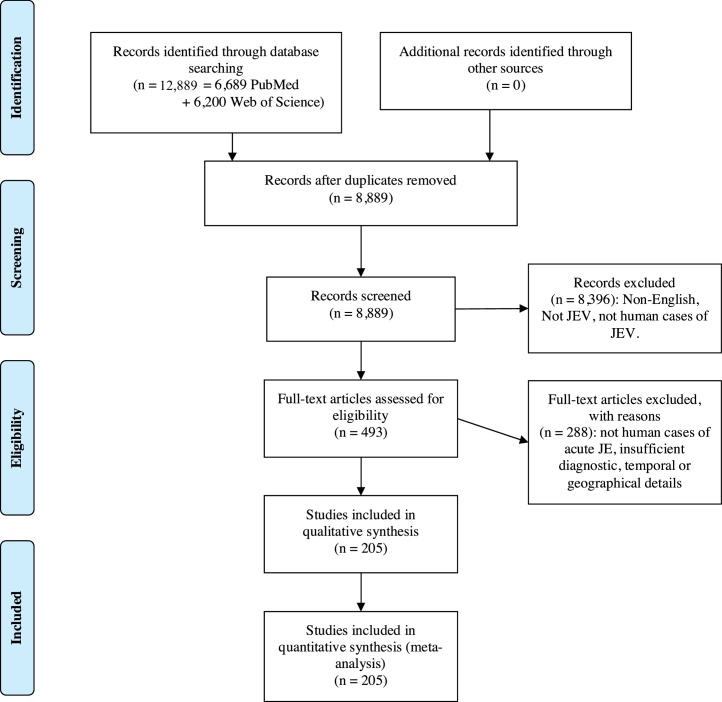
PRISMA flow diagram.

This review identified 205 studies in 22 countries in which a total of 20,212 JE patients were confirmed by laboratory tests; see Figure 2 for the PRISMA flow diagram. Patients were predominantly diagnosed in Asia, with a suggested case of autochthonous transmission diagnosed in Angola. The studies incorporated a variety of methods for the diagnostic tests, including conventional and novel approaches, as summarised in Table 3. The data do not provide evidence of change in the certainty of diagnosis through time, see Table 4.

**Table 3 tbl0015:** Diagnostic methods used for evidence of Japanese encephalitis virus infection.

Diagnostic method	Confirmatory level	Advantage	Disadvantage
Virus detection: Virus Isolation: Inoculation of patient samples into animals Inoculation of patient samples onto primary chick or duck embryo cells, and cell lines including Vero, LLCMK, C6/36, MRC and AP61 Inoculation of patient samples into mosquitoes Virus antigen detection: Reverse passive haemagglutination Immunofluorescence microscopy Staphylococcal coagglutination tests using polyclonal or monoclonal antibodies Monoclonal antibody/immunogold/silver-staining (M-IGSS) ELISA to detect a viral protein (NS1)	Level 1	Direct detection of the virus or viral protein and high specificity Viral isolation provides molecular epidemiological data	Low sensitivity, laborious and viral isolation requires biosafety 3 laboratory capacity
Molecular detection: Conventional RT-PCR Real-time RT-PCR Nested PCR Specific vs. pan-flavivirus Multiplex PCR Next-generation sequencing	Level 1	Direct detection of the viral genome, provides high specificity and additional molecular epidemiological data	Low sensitivity
Antibody detection: Seroneutralization IgM antibody capture ELISA Avidin biotin system Biotin-labeled immunosorbent assay Nitrocellulose membrane-based immunoglobulin M capture dot enzyme immunoassay Haemagglutination inhibition +/- sucrose gradient density centrifugation and 2-mercaptoethanol treatment (2-ME) to detect IgM Complement fixation test Single radial hemolysis	Level 2 for seroconversion demonstrated by neutralization Level 3 for detection of IgM in CSF and for seroconversion or ≥4x rise in Ab titer; Level 4 for detection of Ab detection in a single sample	Good sensitivity Good specificity for primary infection Commercial kit available Good sensitivity	Cross-reaction with other flaviviruses Requires paired samples Laborious Difficult to interpret in secondary infection Limited specificity Cross reaction with other flaviviruses Requires paired samples Difficult to interpret in secondary infection

**Table 4 tbl0020:** Temporal changes in JE diagnostic confirmatory level. Percentage of symptomatic human JE cases reported in the English-language literature in blocks of five years that were confirmed by laboratory testing Level 1-4*.

* A total of 20,212 laboratory-confirmed JE cases were identified. Data are reported according to the year of publication. Inclusion criteria also required geographical (country) and temporal (year) data. Confirmatory levels of JE diagnosis detailed in Table 2; level 1 provides the highest level of confidence, level 3 or 4 refers to cases that were reported as IgM detected in CSF and/or serum.

## Overview of JEV diagnostic testing

The first isolation of JEV was in 1934 when Hayashi demonstrated that a filterable agent inoculated into monkeys produced encephalitis (Hayashi, 1933). The experiment was performed using homogenized brain obtained at post-mortem from a fatally-infected child who presented with encephalitis in Tokyo during the 1924 epidemic. Early studies relied on clinicopathological correlates in infected humans, when compared with those observed following animal inoculation of post-mortem samples. Subsequently, hamster, porcine, and human cell culture systems were developed, which revealed cytopathic effects when inoculated with JEV-infectious specimens (Miyake, 1964, GC-Y et al., 1965). As these procedures improved, mosquitoes and mosquito-cell cultures were added to the resources for isolation and identification of JEV (Gajanana et al., 1996, Johnson et al., 1985). Cerebrospinal fluid (CSF) and other body fluids were also included for analysis (Kumar et al., 1990, Kumar et al., 1994). Subsequently, JEV antigen detection procedures including complement fixation, immunofluorescence microscopy of cells in CSF, reverse passive haemagglutination, and staphylococcal coagglutination (Mathur et al., 1982, Ravi et al., 1989a, Zhang et al., 1989, Mathur et al., 1990) were added to the list of diagnostic tests. Nonetheless, assays involving direct virus detection are minimally useful for the diagnosis of JE as the level of viremia is usually low, and the virus is detectable only briefly early in the infection (Kumagai and Kurokochi, 1950).

In the mid-twentieth century, investigation of the antigenic properties of JEV soon led to the development of serological assays, including complement fixation (Casals and Palacios, 1941a), inhibition of haemagglutination (Clarke and Casals, 1958), and virus neutralization tests (Sabin, 1947, Sever, 1962). Early reports of human infection in 1947 used a seroneutralization technique in which a patient sample was mixed with virus and inoculated into mice (Sabin, 1947, Sabin et al., 1947a, Kuttner and Ts’un, 1936). In 1941, Casals and Palacios published a report on the application of the complement fixation technique (Casals and Palacios, 1941b). The method was used for many years, although it was insensitive, particularly during the acute illness (Rose, 1992). In 1958, Clarke and Casals published a report on the application of the haemagglutination inhibition test (HI) (Clarke and Casals, 1958). The principle exploits the fact that JEV envelope protein agglutinates erythrocytes. Anti-JEV antibodies, developed following infection, bind to JEV protein and thus prevent erythrocyte agglutination, hence the term haemagglutination-inhibition. This remained the method of choice for JE diagnosis, by serological methods, for many years (Okuno et al., 1975, Ding et al., 2007, Barzaga, 1989, Thisyakorn and Nimmannitya, 1985) and was subsequently adapted as a more convenient microtiter method (Gajanana et al., 1996, Sever, 1962, Gunakasem et al., 1981). However, limitations in the sensitivity and specificity of the assay were recognized by Clarke and Casals (Clarke and Casals, 1958). Moreover, the test relies on the combined paired results obtained from acute and convalescent serum samples, thus taking weeks for confirmation (Burke et al., 1987). Other serological methods, such as single-radial hemolysis (Chan et al., 1985, Duca et al., 1979, George and Pavri, 1986), were also introduced. However, inadequacies were readily acknowledged, and it was accepted practice to perform these tests in parallel with others, thus increasing the workload and extending the time for results to be obtained (Buescher et al., 1959, Cardosa et al., 1991).

**Figure 3 fig0015:**
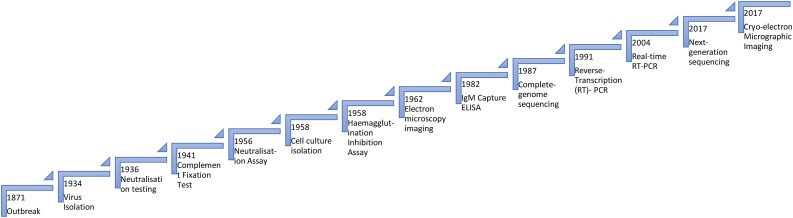
Chronological representation of discoveries related to the detection of Japanese encephalitis virus infection (Miyake, 1964, Sever, 1962, Kuttner and Ts’un, 1936, Casals and Palacios, 1941b, Rose, 1992, Okuno et al., 1975, Casals and Palacios, 1941c, Ravi et al., 1989b).

The plaque-reduction neutralization test (PRNT) was subsequently developed as the gold-standard for JE diagnosis, using paired acute and convalescent sera and comparison with other endemic flaviviruses, and it remains so today (Hills et al., 2009). The demonstration of increasing anti-JEV neutralizing antibody titer in the convalescent serum and the absence, or at least fourfold lower titer for neutralizing antibodies against control-related flaviviruses, provides a robust diagnosis. However, this is laborious, time-consuming, and requires high-level containment facilities for safe manipulation of infectious JEV in cell culture. Mainly for these reasons, the anti-JEV IgM capture ELISA (JEV MAC-ELISA) was developed during the 1980s and has been incorporated as the WHO standard procedure for JE diagnosis (Burke et al., 1982, World Health Organization, 2007). Although commercial JEV MAC ELISAs are manufactured, they may be hard to access in endemic countries (for example, there is no supplier in Laos), relatively expensive, and require costly ELISA readers and significant technical training. The performance of the kit requires specialized laboratory equipment, and thus are by no means point-of-care tests. In addition, field studies suggest that the sensitivity is 50-70% (Robinson et al., 2010), and concerns have been raised regarding the diagnostic specificity (Dubot-Peres et al., 2015). In the last two decades, there has been increased availability of molecular testing, providing crucial data on molecular epidemiology. As well, the aforementioned low and brief viremia limits the role of screening for JEV RNA for diagnostic purposes. Similarly, advances in techniques such as near-atomic resolution cryo-electron microscopy contribute to our understanding of the detailed viral structure, but not to the routine detection of human infection (Figure 3).

## Specific findings of JEV diagnostics review

Studies reporting on the use of seroneutralization, IgM ELISA and RT-PCR are discussed below, since these assays are, at present, the ones most widely incorporated into clinical diagnostics.

Seroneutralization assays: Thirty-two studies identifying evidence of JE using neutralization assays (see Table 5) are included, of which 15 clearly performed tests on acute and convalescent sera, indicating seroconversion (Cardosa et al., 1991, Peiris et al., 1992, CDC, 2005, CDC, 2011, Anga et al., 2010, Anukumar et al., 2014a, Hennessy et al., 1996, Hossain et al., 2010, Langevin et al., 2012a, Lee et al., 2012, Li et al., 2016, Olsen et al., 2010, Ompusunggu et al., 2008, Saito et al., 1999a, Solomon et al., 2008b, Sunwoo et al., 2016, Touch et al., 2009a). These were largely PRNT (14 articles) and/or a microtiter modification in a 96-well plate (4 articles). Three studies performed focus-reduction seroneutralization tests (FRNT), a high-throughput modification of the PRNT involving 96-well plates and an immunocalorimetric assay for end-point determination. Other studies did not describe their methods in detail or cite references to support them. Eleven reported JEV strain used (Sabin (1947); Cardosa et al., 1991; Li et al. (2016); Saito et al. (2015); Benenson et al., 1975a; Ravi et al., 2009; Desai et al. (1997a); Anukumar et al., 2014b; Kyaw et al., 2019; Sabin et al., 1947b; Borah et al., 2011b): they were all genotype 3 viruses, except for one that reported the use of genotype 1 and 3 strains to enable neutralization-based genotype differentiation (Saito et al., 2015). Five studies indicated JEV inoculating dose: the end-point was identified by visual inspection of the cytopathic effect (CPE), staining, or immunofluorescence. All reports appeared to use two-fold dilutions of serum samples, between 1:10 to 1:640 or higher. In terms of quality control, three studies detailed other viruses used, and the use of replicates. Five studies (Sabin (1947); Cardosa et al., 1991; Ravi et al., 2009; Anukumar et al., 2014b; Sabin et al., 1947b) included the use of other flaviviruses such as dengue viruses, West Nile virus, or yellow fever virus.

**Table 5 tbl0025:** Details of seroneutralization testing.

Reference	Country sampling	Country testing	Technique	JEV Strain	Other viruses tested	Cells	Virus Dose	End-point	Samples tested	Algorithm for seroneutralisation
Sabin et al. (1947a)	Republic of Korea	Japan	Mice inoculation	G3 (Nakayama), human brain, Tokyo, Japan, 1935 (EF571853)	NR	Intracerebral and intraperitoneal inoculation in mice	NR	NR	Acute and f/up serum, and CSF	All samples
Sabin et al. (1947b)	China	Japan	Mice inoculation	G3 (Nakayama), human brain, Tokyo, Japan, 1935 (EF571853)	NR	Intracerebral and intraperitoneal	NR	NR	Acute and f/up serum, and CSF	All samples
Sabin (1947)	Japan	Japan	Mice inoculation	G3 (Nakayama), human brain, Tokyo, Japan, 1935 (EF571853)	NR	Intracerebral and intraperitoneal	NR	NR	Acute and f/up serum, and CSF	All samples
Edelman and Pariyanonda (1973)	Vietnam	Vietnam	PRNT	NR	DENV	NR	NR	NR	NR	NR
Benenson et al. (1975b)	Thailand	Thailand	PRNT	G3 strain (Nakayama), human brain, Tokyo, Japan, 1935 (EF571853)	DENV 4	LLC-MK2 cells	50-100 PFU	NR	Acute and f/up serum	NR
Hoke et al. (1988)	Thailand	Thailand	NR	NR	NR	NR	NR	NR	NR	NR
Cardosa et al. (1991)	Malaysia	Malaysia	PRNT	G3 strain (Nakayama), human brain, Tokyo, Japan, 1935 (EF571853)	DENV 2 (16681 strain)	*Aedes albopictus* C6/36 cells	NR	50%*	Acute and f/up serum	Confirmatory testing after positive MAC-ELISA
Peiris et al. (1992)	Sri Lanka	Sri Lanka	Microtitre VNT	NR	NR	Porcine stable (PS) kidney cells	NR	80%*	Serum (NR if acute and/or f/up)	NR
Wittesjo et al. (1995)	Indonesia	Sweden	PRNT	NR	NR	NR	NR	80%*	Acute and f/up serum	NR
Hennessy et al. (1996)	China	U.S.A.	PRNT	NR	NR	NR	NR	NR	CSF, Acute and f/up serum	All samples
Desai et al. (1997b)	India	India	Microtitre VNT	G3 (P20778/P20), human brain, Vellore, India, 1958 (AF080251)	NR	Porcine stable (PS) kidney cells	100 TCID 50	100%*	CSF	All samples
Saito et al. (1999c)	Japan	Japan	FRNT	NR	YFV	BHK-21 cells	NR	50%*	CSF, Acute and f/up serum	All samples
Tiroumourougane et al. (2003)	India	India	NR	NR	NR	NR	NR	NR	NR	NR
Cutfield et al. (2005)	China	New Zealand	NR	NR	NR	NR	NR	NR	Acute and f/up serum	NR
CDC (2005)	Thailand	U.S.A.	NR	NR	NR	NR	NR	NR	Acute and f/up serum	NR
Ompusunggu et al. (2008)	Indonesia	Indonesia	PRNT	NR	NR	NR	NR	NR	Serum (NR if acute and/or convalescent)	NR
Lehtinen et al. (2008)	Thailand	Finland	PRNT	NR	DENV 2	NR	NR	NR	Acute and f/up serum	NR
Ravi et al. (2009)	India	India	PRNT	ChimeriVax™-JEV	ChimeriVax™-DENV 2	Vero cells	NR	NR	CSF	Confirmatory testing after positive or equivocal MAC-ELISA
Touch et al. (2009b)	Cambodia	Cambodia	PRNT	NR	NR	Vero cells	NR	NR	NR	NR
Anga et al. (2010)	Papua New Guinea	Australia	PRNT	NR	NR	NR	NR	NR	NR	NR
Hossain et al. (2010)	Bangladesh	U.S.A.	PRNT	NR	NR	NR	NR	90%*	NR	NR
CDC (2011)	U.S.A. (Travellers from the Philippines and Thailand)	U.S.A.	NR	NR	NR	NR	NR	NR	CSF	NR
Borah et al. (2011b)	India	India	Microtitre VNT	G3 strain (P20778/P20), human brain, Vellore, India, 1958 (AF080251)	NR	BHK-21 cells	100 TCID50 in 50 μL	50%*	Acute and f/up serum	Patients with paired serum available after MAC-ELISA tested
Lee et al. (2012)	Republic of Korea	Republic of Korea	NR	Not reported	NR	NR	NR	NR	Acute and convalescent serum	Confirmatory testing after positive MAC-ELISA/HI/IIF.
Langevin et al. (2012b)	Canada (Traveller from Thailand)	Canada	NR	NR	WNV and DENV	NR	NR	NR	CSF, Acute and convalescent serum	All samples
Hills et al. (2014)	China, Taiwan, Republic of Korea	U.S.A.	NR	NR	NR	NR	NR	NR	Acute and f/up serum	NR
Anukumar et al. (2014b)	India	India	Microtitre VNT	G3 (P3), human brain, Bankura, India, 1973 (AB379813/Z34095)	WNV	Porcine stable (PS) kidney cells	100 TCID50	50%*	Acute serum	All acute serum
Rayamajhi et al. (2015)	Nepal	U.S.	PRNT	NR	DENV, WNV, and Powassan viruses.	NR	NR	NR	NR	Confirmatory testing after positive or equivocal MAC-ELISA
Saito et al. (2015)	Laos	Japan	FRNT	Nakayama (a pathogenic and vaccine strain, Tokyo, Japan, human brain, 1935, G3), Beijing-1 (a pathogenic and vaccine strain, Beijing, China, human brain, 1949, G3), P19-Br (an isolate, Chiang Mai, Thailand, human brain, 1982, G1), LaVS56 (an isolate, Vientiane, Lao PDR, swine sera, 1993, G1), and LaVS145 (an isolate, Vientiane, Lao PDR, swine sera, 1993, G1)	DENV 1 (Hawaiian), 2 (New Guinea B), 3 (H-87), and 4 (H-241) and WNV	BHK-21 cells	NR	50%*	Acute and f/up serum	All samples
Li et al. (2016)	China	China	PRNT	G3 strain (733913), human brain, Beijing, China, 1949 (AY243805/AY243844)	NR	BHK-21 cells	100 PFUs	90%*	Acute and f/up serum	All serum
Sunwoo et al. (2016)	Republic of Korea	Republic of Korea	NR	NR	NR	NR	NR	NR	NR	NR
Kyaw et al. (2019)	Myanmar	Myanmar	FRNT and PRNT	G3 strain (JaOrS982), mosquitos, Japan, 1982 (NC_001437)	DENV 1-4	NR	NR	NR	CSF	NR

G1 and 3 = genotype 1 and 3; NR = not reported; CSF = cerebrospinal fluid; PRNT = plaque reduction neutralization test; DENV = Dengue virus; VNT = viral neutralization test; FRNT = focus reduction neutralization test; TCID = median tissue culture infectious dose.

* titer required to reduce dengue viral plaques/focus/CPE by 50%, 80%, or 90%. MAC-ELISA = IgM antibody capture enzyme-linked immunosorbent assay, HI = haemagglutination assay, IIF = Indirect immunofluorescence assay.

Studies followed different algorithms for including neutralization in patient testing, but it was primarily performed to confirm equivocal cases in other serological tests. Acute and/or follow-up serum and/or CSF were tested. For studies that did report individual results, confirmation was rarely achieved as there was either insufficient serum, failure to detect a four-fold rise of antibody titer in the convalescent serum, or cross-reactivity was detected with related viruses included as controls in the tests.

IgM ELISA: One hundred and sixty-three (80%) studies reported the results of tests using IgM MAC-ELISA methods. Notably, 115 of these studies tested both CSF and serum samples and presented results for the different body fluids separately. One hundred and twenty-two (74%) reported the method, of which 66 (40%) used in-house methods, and 33 (20%) used commercial kits. The primary in-house methods involved those described by Burke et al. (1982), Innis et al. (1989), the National Institute of Virology, Pune (Prasad et al., 1993). Commercial kits were purchased from PanBio (Touch et al., 2009b), Venture Technologies (Cardosa et al., 2002), XCyton Diagnostics Ltd. (Borthakur et al., 2013), and Shanghai B&C Biological Technology Co. Ltd. (Feng et al., 2013). There was minimal reporting of quality control measures such as control specimens and repeat testing of positives; 46 (28%) reported following the manufacturer’s instructions. In total, 7,584 JE patients (38%) were diagnosed by MAC-ELISA in serum and/or CSF, i.e., results for the different body fluids were not reported separately, with 3,668 (18%) positive in CSF alone. Ninety-one (56%) studies using MAC-ELISA also reported testing for dengue virus infection to confirm specificity for JEV, i.e., they were not cross-reactive with dengue viruses.

Molecular tests: Forty-one studies (25%) reported the use of reverse transcription-polymerase chain reaction tests (RT-PCR), of which 13 (68%) described the methods used or cited corresponding references. These targeted various regions of JEV genome, including the capsid (C), pre-membrane (prM), envelope (E), non-structural (NS) proteins NS2A, NS3, NS5 and the untranslated regions (UTR). These studies reported the use of the conventional RT-PCR standard test, with nested or hemi-nested techniques, and real-time techniques (RT-qPCR) using hydrolysis probes or SYBR green. The studies reported the use of conventional RT-PCR either standard, with nested or hemi-nested techniques, and the real-time techniques (RT-qPCR) using hydrolysis probes or SYBR green. In total, 332 (1.7%) patients were positive when tested by RT-PCR.

Other tests: Fifty-eight (28%) studies reported the isolation of JEV *in vivo* or *in vitro*. Forty-two (20%) performed HI, 14 (7%) performed complement fixation, and seven (4%) performed indirect immunofluorescence assays. Two (1%) studies diagnosed cases by next-generation sequencing.

## Discussion

This review reveals that current JE diagnostic techniques are confined mainly to those with a low confidence level, i.e., anti-JEV IgM detected in serum samples, or in which reported results do not differentiate between detection of anti-JEV IgM in CSF and serum. There is no doubt that the introduction of IgM ELISA testing in serum samples represents real progress. However, we are now much better informed about the limitations of relying on this method of detection. We also acknowledge as a limitation that the studies included in this review were performed in different settings, with different constraints, financial limitations, and resources available. Nonetheless, we highlight the need for both an improvement in the accuracy of routine laboratory diagnostics, and also the development of point-of-care tests to confirm cases in JE endemic areas that frequently have no laboratory capacity. Below, we discuss the existing assays in more detail.

Seroneutralization tests: Seroneutralization is considered the gold standard for the diagnosis of infections due to pathogenic viruses such as JEV, but in the published literature cited here, it was only performed in approximately 16% (32/205) of studies as laboratory confirmation. This is probably because performing seroneutralization is technically demanding and requires sufficient volumes of serum/CSF to enable the inclusion of control viruses and duplicates of each titration. Since JEV is a human pathogen with high individual risk, seroneutralization has to be performed in a biosafety level 3 laboratory, placing additional burdens on time, cost, and qualified personnel. Another potential complication may arise when sera from patients who have previously been exposed to JEV-related flaviviruses may contain higher titers against the closely related flaviviruses than against JEV (“doctrine of original antigenic sin”) (Francis, 1960). For example, the titers of anti-YFV neutralizing antibodies were higher than anti-JEV neutralizing antibodies in JE patients who had previously received the yellow fever vaccine (Saito et al., 1999c).

Similarly, in a study testing for West Nile virus and JEV, 18 patients’ data remained equivocal due to high levels of antigenic cross-reactivity between these viruses (Anukumar et al., 2014b). The neutralization test may only be strictly applicable as the gold-standard for vaccine efficacy studies when a baseline serum sample is compared with a convalescent sample taken at a fixed interval 1-3 months later. To confirm acute JEV, neutralization is an imperfect gold standard. Severe constraints on being able to arrange for sample testing by neutralization, and the results being interpretable without cross-reactive positivity due to other flaviviruses (which is relatively rare in JEV endemic areas), impede 'neutralization confirmation.' The neutralization titers obtained may be affected by the particular strain of challenge virus utilized (Ferguson et al., 2008). A final issue with the neutralization test is the inability to detect non-neutralizing antibodies, thus potentially reducing the analytical sensitivity (Johnson et al., 2016). Therefore, the practicalities of PRNT and diagnostic yield when testing field samples can be low, although the specificity is potentially high.

IgM ELISA: Anti-JEV IgM detection by MAC-ELISA is the WHO recommended standard diagnostic test, and 80% (163/205) of studies reported the use of a MAC-ELISA. It is recognized that JEV diagnosis by testing CSF provides considerably more reliable confirmation than the use of serum (Granerod et al., 2010a). However, obtaining acute and convalescent CSF and serum samples can be difficult, particularly in rural Asia where access is logistically tricky, and personnel and appropriate facilities are limited. Only 91 (56%) studies reported contemporaneous testing for anti-dengue-specific antibodies. There are issues in the accurate full reporting of results, both for the breakdown of which patients were diagnosed by testing CSF and/or serum, and contemporaneous testing for dengue-specific antibodies.

Reverse-transcription polymerase chain reaction assays (RT-PCR) for the detection of JEV RNA:

Diagnosis by the detection of the viral genome by DNA amplification generated by RT-PCR is a valuable addition to diagnostic procedures for RNA viruses. The test has high analytical sensitivity, is very specific, and can provide additional information that can be exploited to understand the molecular epidemiology of the detected virus. Nonetheless, JE cases are rarely confirmed (1.7% in this review) using RT-PCR technology, although this will undoubtedly increase in usage as point-of-care and automated methods are developed. Poor reporting of the techniques used in many publications hinders our ability to make comparisons of the efficacy of different methods (see Bharucha et al. (2018a). There does appear to be higher analytical sensitivity in studies that used nested and hemi-nested techniques as compared to those using single RT-PCR; however, these techniques are notoriously prone to contamination, causing false-positive results. It is also recognized that the sensitivity of nucleic acid (and protein) detection will continue to increase as technology improves (Bai et al., 2018, Zang et al., 2019). Evidence to suggest that this will be the case arises from the high cycle threshold (Ct) of patients that are confirmed, and the fact that blood donor transmission has been seen in WNV patients who were negative by RT-qPCR tests (Dodd et al., 2015). The recent detection of JEV RNA in throat swabs of JE patients suggests that this non-invasive procedure may marginally improve diagnostic yield (Bharucha et al., 2018b). There have now been two cases of JE, confirmed by RT-PCR, that were first identified by metagenomic next-generation sequencing (mNGS) (Simon-Loriere et al., 2017b, Mai et al., 2017); the first detection of JEV RNA in human urine, and JEV detection in serum from an African patient with a co-YFV infection. The latter was not confirmed by an orthogonal method and remains questionable. Nonetheless, unbiased mNGS technology (see below), application, and reporting will continue to improve, and could potentially detect JEV in new locations (Brown et al., 2018).

Requirements of a new test for the detection of JEV infection

CNS infections are challenging syndromes to diagnose and treat, even in the most highly resourced centers (Kelly et al., 2012, Solomon et al., 2012). It is estimated that they may be caused by >100 different pathogens, including novel and emerging pathogens (Granerod et al., 2010b). Current approaches to diagnosis in routine clinical practice involves targeted strategies, suggesting that some (potentially treatable) infectious aetiologies are missed (Brown et al., 2018). Clinical diagnosis is rarely absolute, and confirmation requires access to appropriate laboratory facilities and personnel, lacking in many areas worldwide (Wilson et al., 2018). While the analysis of brain biopsy material is the gold standard, it is not possible in most cases. Aetiological diagnosis usually involves an invasive lumbar puncture (LP) to obtain CSF, which in turn requires the appropriate clinical skills, infrastructure, and patient acceptability. Diagnostic assays are frequently difficult to interpret, may demonstrate poor accuracy and poor discrimination between previous vaccination or non-neurological JEV infection (Granerod et al., 2010a). Targeted research is needed to raise the bar for both the improvement in laboratory diagnostics as well as the development of point-of-care tests (John et al., 2015).

In clinical and epidemiological situations, the detection of JEV RNA can provide an invaluable indication of infection. The sensitivity of this test is, to a large extent, limited by a combination of the short period of viremia, the relatively low concentration of virus in CSF, and the fragility of RNA. The introduction of highly sensitive point-of-care tests that may be used to analyze multiple body fluids in parallel would partly resolve these challenges. Recently, there have been significant developments in highly sensitive molecular point-of-care tests for flaviviruses, such as reverse transcription loop-mediated isothermal amplification (RT-LAMP), reverse transcription recombinase polymerase amplification (RT-RPA), nucleic acid sequence-based amplification (NASBA), transcription-mediated amplification (TMA), helicase-dependent amplification (HDA), and nicking enzyme amplification reaction (NEAR) (Calvert et al., 2017, Ganguli et al., 2017, Kurosaki et al., 2017, Mauk et al., 2017, Priye et al., 2017, Yaren et al., 2018, Castro et al., 2018, Guo et al., 2018, Kim et al., 2018a, Kumar et al., 2018, Lopez-Jimena et al., 2018, Lamb et al., 2018, Sabalza et al., 2018, Song et al., 2018, Zhao and Feng, 2019, Abd et al., 2017, Chan et al., 2018, Tan et al., 2018, Vasileva Wand et al., 2018, Saa et al., 2018). Microfluidics, chips, paper-based devices, and biosensors are also being developed (Onyango et al., 2017, Adegoke et al., 2017, Afsahi et al., 2018, Ariffin et al., 2018, Wasik et al., 2017).

For the time being, we will need to rely on serology for diagnostic confirmation. During the past three years, with the international focus on emerging flaviviruses following the chikungunya virus and theZika virus global epidemics, there have been intensified efforts to reduce cost, increase throughput, and improve specificity. These include the analysis of IgA (Amaro et al., 2019, Warnecke et al., 2019, Colonetti et al., 2018, Nascimento et al., 2018, Rockstroh et al., 2017, Zhang et al., 2017, Huang et al., 2017, Balmaseda et al., 2008, Balmaseda et al., 2003, Yap et al., 2011) and IgG subclasses (Nascimento et al., 2018), antibody avidity (Amaro et al., 2019, de Vasconcelos et al., 2018, Ronnberg et al., 2017, Tsai et al., 2018, Shen et al., 2017), incorporation of blocking agents, IgG depletion (Calvert et al., 2018) and production of specific monoclonal antibodies for identification of specific viral epitopes (Zhu et al., 2018, Lebani et al., 2017, Piyasena et al., 2017, Kim et al., 2018b, Frietze et al., 2017). This recent work highlights the inherent challenges of serological techniques for JE identification. As Lindsey et al. describe, antigenic cross-reactivity between related viruses can make it virtually impossible to distinguish the cause of the infection (Lindsey et al., 2018). For example, cross-reactive IgM class antibodies may not be stimulated during a related secondary flavivirus infection. On the other hand, IgA may be produced during a secondary flavivirus infection, and a laboratory-defined ‘seroconversion’ might be detected following a secondary flavivirus infection by a related flavivirus.

Evidence suggests that the secreted viral JEV non-structural protein 1 (NS1) is present at very low concentrations in serum or CSF, unlike in dengue (Li et al., 2012, Kumar et al., 2011). A novel alternative approach would be to analyze the host response, using transcriptomics or proteomics. However, questions of specificity and how these would be translated into point-of-care tests will require detailed investigation and the development of innovative methodologies.

In summary, while the diagnosis of JE has been possible for many years, it still requires specialized high-containment laboratories and appropriately trained scientists. Therefore, it cannot be reliably carried out in many resource-limited regions where JEV is endemic/epidemic. A fundamental pre-requisite in the public health strategy for the control of JE is lacking, that of a reliable and simple diagnostic procedure that can be adapted for point-of-care tests, and readily available for use throughout JEV endemic regions of the world. Improved diagnostic capabilities throughout JEV affected areas will not only benefit individual patients (through accurate diagnosis) but lead to higher quality surveillance data and a better understanding of the distribution of JE risk, enabling improved targeting and evaluation of interventions. The lack of diagnostic capabilities for JE is a barrier to understanding the actual disease burden and the impact of public health strategies.

## Ethical approval

Ethical approval was not required for this study.

## Conflict of interest statement

None of the authors have any conflict of interest to report.

## References

Team RDC, 2010, Saito et al., 1999b, Borah et al., 2011a.
